# Root causes for delayed hospital discharge in patients with ST-segment Myocardial Infarction (STEMI): a qualitative analysis

**DOI:** 10.1186/s12872-015-0105-2

**Published:** 2015-09-29

**Authors:** Jeremy Adams, Brian Wong, Harindra C. Wijeysundera

**Affiliations:** Division of Cardiology, Schulich Heart Centre, Sunnybrook Health Sciences Centre, 2075 Bayview Avenue, Suite A202, Toronto, ON M4N3M5 Canada; Department of Medicine, Sunnybrook Health Sciences Centre, University of Toronto, Toronto, ON Canada; Centre for Quality Improvement and Patient Safety, University of Toronto, Toronto, ON Canada; Institute of Health Policy, Management and Evaluation, University of Toronto, Toronto, ON Canada; Institute for Clinical Evaluative Sciences (ICES), Toronto, ON Canada; Li Ka Shing Knowledge Institute of St. Michael Hospital, University of Toronto, Toronto, ON Canada

**Keywords:** Zwolle Score, STEMI care, Qualitative analysis

## Abstract

**Background:**

The majority of patients who suffer a ST-segment myocardial infarction (STEMI) are hospitalized for longer than 48 h. With the advent of reperfusion therapy, the benefits of such extended hospitalization has been questioned. The goal of this qualitative study was to identify the root causes for prolonged hospitalization in STEMI patients in order to refine future interventions to optimize the length of hospitalization.

**Methods:**

Practitioners involved in the discharge process for STEMI patients at a single tertiary care STEMI center underwent semi-structured interviews focused on three fictional patient cases. Data were transcribed and analyzed for key themes by thematic analysis.

**Results:**

Interviews were conducted with 17 practitioners (5 Attending Physicians, 4 Internal Medicine Residents, 4 Cardiology Residents, 4 Nursing Staff). The key themes were patient factors, provider factors, and transitions to outpatient care. Patient factors included concerns that early discharge would limit dose titration of medications, the educational experience of the patient, and prevent monitoring for complications. Provider factors included past clinical experience with STEMI complications, in turn impacting discharging behaviour. Transitions of care factors were difficulty in establishing reliable follow-up plans and home care services.

**Conclusions:**

Several themes were identified that influence the timing of discharge post STEMI. The majority of these issues are not incorporated into currently available post STEMI risk stratification tools. Future quality improvement interventions to reduce STEMI length of stay should focus on in-patient and out-patient strategies to address these unique clinical situations.

**Electronic supplementary material:**

The online version of this article (doi:10.1186/s12872-015-0105-2) contains supplementary material, which is available to authorized users.

## Background

ST-segment myocardial infactions (STEMI) are caused by an acute total occlusion of an epicardial coronary vessel, and remain a significant cause of hospitalization in Canada [1, 2]. Published STEMI guidelines provide in-depth recommendations of pre-hospital, hospital-based, and post-hospital care and interventions for patients with STEMI [3]. Before the introduction of reperfusion therapy, length of stay was traditionally between 7–10 days [4]. This time in hospital was necessary for medication up titration, monitoring for arrhythmia, and patient education. With the development of modern reperfusion therapies, however, the benefit of an extended hospitalization has been questioned [5].

A study of 23,000 STEMI patients found that keeping patients beyond a third day in hospital was not cost effective [6]. This finding prompted the derivation of the Zwolle risk score to estimate early mortality risk, using data from 1794 patients who were treated with primary angioplasty from 1994 to 2001 [5]. The goal of this score was to identify patients who may be safe for early discharge after receiving contemporary STEMI care. The Zwolle score incorporates clinical variables such as presence of heart failure, location of the infarction, patient age, success of reperfusion therapy and the angiographic extent of underlying coronary disease (see Additional file 1: Appendix 1 for scoring details) to provide estimates for mortality at 0–2 days, 2–10 days, and 30 days. At a score of less than or equal to 3, mortality after 2 days was extremely small at 0.2 %. The Zwolle investigators argued these very low risk patients could be discharged from hospital between 48–72 h after STEMI and that substantial cost savings could be realized as a result.

Prior studies have investigated protocol-based interventions to reduce length of stay in hospital after STEMI. In 1988, Topol and colleagues randomized a group of 90 STEMI patients post reperfusion therapy to early discharge (within 3 days) or usual care [4]. These were patients with no obvious STEMI complications and a low risk exercise stress test. At 6 months of follow-up, there was no significant difference in hospital re-admissions or complications in the early discharge group. The second Primary Angioplasty in Myocardial Infarction (PAMI-II) trial randomized low risk patients (age < 70 years, left ventricular ejection fraction >45 %, one or two vessel disease, successful reperfusion, no persistent arrhythmias) to discharge on day 3 versus usual care with pre-discharge exercise testing [7]. The patients randomized to discharge on day 3 had similar rates of mortality, re-infarction, congestive heart failure, and stroke as patients randomized to usual care.

Despite such evidence, there has been minimal adoption of early discharge into routine clinical practice [8]. Pilot work from our institution showed that of the 1262 patients who were treated for STEMI at Sunnybrook Health Sciences Centre from 2007 to 2012, 1040 patients had low risk Zwolle scores. However, 75 % of these patients had lengths of stay greater than 48 h. These findings are consistent with those observed by other groups. A recent retrospective study of 255 STEMI patients at a Canadian academic hospital found that 72 % of low risk patients had lengths of stay that extended beyond 2 days [8]. Accordingly, the objective of this quality improvement project was to conduct a qualitative analysis to explore the root causes of prolonged post-STEMI at Sunnybrook to inform future interventions designed to reduce length of stay and subsequent hospitalization costs. The failure for risk scores to be successfully translated into changes in clinical practice extends beyond cardiology and the care of STEMI patients. As such, understanding the root causes of prolonged hospitalization and how these may or may not be potentially addressed by current risk scores will have broad implications on the development of new scores and how they are implemented in practice.

## Methods

According to the policy activities that constitute research at Sunnybrook Health Sciences Center, this work met criteria for operational improvement activities exempt from institutional research board review.

Using the Model for Improvement as the theoretical basis for our study, our qualitative analysis intended to better characterize the nature of the QI problem at hand in order to answer the question: “What changes can we make that will result in improvement?” [9] Our goal was to have our results serve as the template for developing a quality intervention focused on increasing the proportion of low risk STEMI patients discharged within 72 h.

Our primary study was a qualitative analysis of discharge practices generated from semi-structured interviews with practitioners at Sunnybrook’s Coronary Intensive Care Unit (CICU) and cardiology ward. [10] Qualitative data has previously been used to investigate processes of care in STEMI patients. Bradley and colleagues used qualitative methods to describe the characteristics of high performance hospitals in achieving optimal door to balloon times for primary PCI [11]. These investigators argued that qualitative data were essential for understanding variation in the complex clinical processes involved in providing primary PCI to a STEMI population. We believe that discharging STEMI patients from hospital represents a similar set of complex clinical processes and is well suited to a qualitative approach. We were interested in the clinical and system factors that influenced individual practitioner’s discharging behaviour. We were particularly interested in understanding why a particular patient with a low risk Zwolle score (less than 3) might not be discharged early from hospital.

Qualitative data were generated using semi-structured interviews with practitioners involved in the front line care of STEMI patients. Interviews were structured around three fictional patient cases designed to illustrate a spectrum of risk according to the Zwolle score (see Additional file 1: Appendix 2 for list of cases). All study participants were asked to comment on the three clinical cases. Follow-up questions and discussion occurred around each case. The nature of the discussion around each clinical case was not scripted. Study participants were allowed to raise new themes and clinical scenarios related to STEMI care. A combination of convenience and purposive sampling was used to select interview participants [12, 13]. We attempted to include a representative sample of health care disciplines that are involved in the discharge process: attending physicians, medical and cardiology residents, and nursing staff involved in the discharge process (nurse practitioners and charge nurses). Staff were approached individually to participate. Given that this study was performed by a single investigator, it was not possible to approach all individuals at Sunnybrook Health Sciences centre who were involved in STEMI care.

Interviews were audio recorded and transcribed by a single investigator (JA). The same investigator carried out a thematic analysis of the data using a constant comparative approach to generate high-level themes. These themes or “codes” were used to analyze new data as they are required. As new data were analyzed, codes were adjusted and subcodes created [12, 13]. No major changes were made to the clinical cases of the semi-structured interviews during data collection and analysis. Themes were generated from the transcribed data with inductive reasoning. Data collection and analysis continued until no new themes are generated, at which point saturation was reached.

## Results

Qualitative interviews were conducted with 17 practitioners connected with STEMI care at Sunnybrook. For a summary of disciplines represented, please see Table 1.

**Table 1:** Interview participants

Discipline	Number of participants
Attending physician	5
Medical residents	4
Cardiology residents	4
Nursing staff	4
Total	17

### Thematic analysis of qualitative data

The themes generated from our qualitative data were organized into the following categories: patient factors, provider factors, and transitions to outpatient care (Table 2).

**Table 2:** Association between identified themes and category of interviewee

Theme	Category of participants who emphasized this theme
Medical Care	Attending physicians, residents
Post Myocardial Infarction Education	Nursing staff, residents
Post Myocardial Infarction Complications	Attending physicians, residents
Age/Functional Capacity	Attending physicians, nursing staff
Provider Factors (previous physician experience)	Attending physicians, nursing staff
Transitions to out patient care	Nursing staff, residents

### Patient factors

1. Medical Care

Most individuals interviewed discussed the specifics of providing medical care for patients post STEMI. Many practitioners expressed concern about not achieving maximal doses of medications in the post STEMI setting or the necessary diagnostic testing before sending a patient home. It was also acknowledged that a new medical diagnosis is occasionally uncovered post STEMI that requires an extended stay to investigate and treat.

### Quotations

“…Depends when the echo [cardiogram] was done as well....If the echo wasn’t done, then definitely not. I would wait for a formal echo. -Cardiology Resident

…so sometimes he’s not as clean as you think he is…when the HbA1c comes back, you have to start him on a total new medical regimen **-**Nursing staff ”

2. Post MI Education

Many participants, especially the nursing staff and residents interviewed, emphasized the importance of post MI education. There was concern that reducing the length of stay in hospital may compromise the patient’s understanding of their medical condition and compliance with medical and lifestyle therapy.

### Quotations

“…how well do these patients know their meds and feel comfortable following it, is that preparation done well enough for that somewhat early discharge?…-Nursing Staff

To me if it is a patient that can be on top of his meds and everything and he gets his follow-up seriously…If he is a retired person who has no issues at home, I would say its fine (early discharge)- Nursing Staff”

3. Risk of Complications

It was widely acknowledged amongst the attending physicians and residents interviewed that the primary purpose for hospitalization after STEMI was for the detection and prompt treatment of complications. These complications could be related to the myocardial infarction or the subsequent treatment. When discussing stability for discharge, it was highlighted that lack of STEMI complications was a criteria for discharge. Larger infarctions generally made participants apprehensive about early discharge.

### Quotations

“No clinical bleeding, no Right ventricular infarct, no mitral regurgitation, all the usual suspects that usually come with an inferior STEMI. And then no complications related to the PCI.- Attending Physician.

The one thing that I would want to know is how big his CK rise is. **–** Attending Physician

The only thing I would like to see would be his peak CK. Just to get an idea of how much muscle…In terms of his risk of mechanical complication afterwards.-Cardiology Resident”

The prevention of thromboembolic complications after STEMI garnered much discussion amongst the attending physicians (*n* = 5). The advent of dual antiplatelet therapy in post STEMI care has made the initiation and maintenance of anticoagulation in this setting a more nuanced and complicated process [3]. This primarily applies to patients at risk of left ventricular thrombus or a history of atrial fibrillation. While most study participants favoured anti-coagulation for prevention of LV thrombus and subsequent embolization after anterior infarction, there was significant variation in the enthusiasm for this intervention. Moreover, this was often cited as a reason to delay discharge in patients with anterior infarction. Kotowzcz et al. have demonstrated that patients started on anticoagulation have longer hospitalizations after STEMI [8]. According to the recent STEMI guidelines, anticoagulation with a vitamin K antagonist in patients with anteroapical akinesis or dyskinesis is a class IIb recommendation, indicating that evidence is uncertain and potentially harmful [3]. While there is no clear evidence of benefit in STEMI patients treated with PCI and modern antiplatelet agents, our qualitative data suggests the presence of significant practice variation when physicians are caring for patients with anterior infarctions.

### Quotations

“Ant. STEMI (akinetic wall)…I would generally give them warfarin for the duration, although she is 88 years old. If it is an expanding akinetic apex, I would generally use triple therapy…-Attending Physician

She’s not anti-coagulated. No, I wouldn’t send her home today. She has an akinetic distal anterior wall and apex. She’s on dual anti-platelet therapy. So her highest risk of embolization if she does develop a thrombus would be in the first week post MI, so I actually would have anticoagulated her and made sure she was actually stable on anticoagulation and not bleeding clinically. - Attending Physician

I would send her out on dual antiplatelet. If the CK rise was more than 2500 then I probably would end up getting an echo and then an echo a week after the fact, just to make sure there is no suspected clot there. I’m assuming that it says here, grade 2 LV function, so I’m assuming that they did an echo at some point.... given that she is 88 years old (I prefer not to (anticoagulate).- Attending Physician

Do you anti-coagulate these guys? Grade 2 I don’t. If its in the grade 3/4, then the contrast study can be very helpful if there is any clot there. If its grade 3 I’d prefer to not anticoagulate, grade 4 I would anticoagulate.- Attending Physician”

4. Age and functional capacity

Patient age played a role in participants’ enthusiasm for early discharge. It was acknowledged that elderly patients were at higher risk for complications. Moreover, participants were concerned that some elements of post STEMI care, such as anticoagulation, were substantially more complicated in elderly patients. This often would increase the length of stay that participants would keep patients in hospital.

### Quotations

“I would want to anticoagulate her and make sure she is not bleeding clinically, prior to assessing her falls risks. If she is a good 88 year old I would, if she is not a good 88 year old I wouldn’t.- Attending Physicians

…they are 90 years old, they are not going to go out…most times, right?- Nursing Staff

There is no evidence of heart failure, but just given the fact that it is an anterior STEMI and she is 88.- Attending Physician

This one I’m holding because its anterior STEMI and old. - Attending Physician”

### Provider factors

The influence of previous physician experience was discussed in a number of contexts by one of the attending physicians and members of the nursing staff. It was acknowledged that previous experience and personal beliefs play a significant role in clinical decision-making. As an example, a previous experience with a stroke complicating anterior myocardial infarction will make a physician more likely to recommend anticoagulation. Study participants hypothesized that older physicians may be more conservative in their discharging behaviour because of the era in which they trained and having witnessed a greater number of post discharge complications.

### Quotations

“..the number of times that we have a post MI ventricular dysrythymia is pretty low, so I mean, my tendency to keep them a little longer, I’ve seen more complications and I have started practicing in the era before primary angioplasty…people would keep infarcts in for a week.- Attending Physician

But it is all tempered by experience....when I was a medical student there was a patient at the counter of the nursing station being discharged after anterior infarct, and he had a big stroke in front of my eyes…I wish we had anti-coagulated that guy- Attending Physician

…or the physician, covering the ward…their preference, sometimes they feel comfortable that someone will stay an extra day, everyone is different- Nursing Staff “

### Transitions to out-patient care

The ability to arrange timely support services for elderly patients with functional concerns was also discussed as a factor that would increase length of stay post STEMI. This issues was discussed primarily amongst the nursing staff interviewed. Practitioners, especially the nursing staff and residents, were concerned about the ability of to secure timely follow-up.

### Quotations

“I think in this hospital a big issue is that we don’t identify patients who may need help at home early enough, so we wait, wait…once a patient gets admitted, it should be identified this patient if they are going to go home, cannot live on its own…at least get social work or OT involved…our problems are that we wait, we wait, when you want to talk about it later, but that later…it causes at least 2–3 days…they is no guarantee there is a bed for this patient in rehab, there is not guarantee, so I think we really do poorly in that matter- Nursing Staff

…you’re lucky to get follow-up in 6–8 weeks, its not 4 weeks any more.-Nursing staff

…maybe just one more day and then home. Because out there in the community they don’t really change medications, so maybe we would want him on a higher target dose for his benefit.- Nursing Staff

I don’t really expect them to have any follow-up for a month at least. The family doctors will not change anything. The next time it will be changed is when they see their cardiologist (whenever that is)-Internal Medicine Resident”

## Discussion

This project was a locally based quality improvement initiative to understand the root causes of prolonged lengths of stay for post STEMI patients at a single tertiary care STEMI centre. Qualitative analysis was performed on interviews with 17 front line staff involved in STEMI care at Sunnybrook. Various themes were identified that influence a patient’s length of stay and were organized into the following categories: patient factors, provider factors and transitions to outpatient care. Importantly, the majority of these themes identified issues that were not components of the Zwolle risk score used to identify potential patients for early discharge.

Despite published data supporting the safety of early discharge, the real world application of this strategy remains a challenge [8]. The goal of our qualitative analysis was to provide new insights into the discharge process of STEMI patients that will inform the development of further interventions to reduce length of stay and improve overall quality of care. Our data indicates that the reasons for increased length of stay are not likely a result of a knowledge gap regarding which patients are low risk after STEMI. While the Zwolle score may be effective at identifying patients at low risk of death, the score does not address many of the common concerns and clinical dilemmas faced by practitioners at the time of discharge. These concerns include the availability of adequate follow-up, medication compliance, and the safety of anti-coagulation in contemporary STEMI patients treated with PCI. Amongst practitioners at Sunnybrook, there was a general awareness of the impact of advanced age and frailty on the feasibility of early discharge. It was apparent that a low risk Zwolle score alone in these patients did not give practitioners enough re-assurance to aggressively pursue early discharge. Previous physician experience and personal experience with complications played a significant role in their discharging decisions. We infer from these observations that calculated risk scores alone may be insufficient to trump the powerful effect of witnessing a prior complication. Our research adds to the field by highlighting the potential shortcomings of using a risk score in isolation to promote practice change. Indeed our data suggest that in order for a risk score to be useful, it must address the factors that drive a practitioner’s clinical decision. These findings suggest that work such as ours should be done, prior to the development of a risk score using traditional statistical methods.

We propose that systems of care be developed that address the clinical issues raised by our qualitative data. Concerns about medication adherence, patient education and length of time from discharge to follow-up may be mitigated by early visits to dedicated STEMI clinics. While potentially resource intensive, this approach may prove less costly than extra days in hospital. Improved systems of communication with primary care physicians, electronically or via telephone, should be trialed and evaluated. Targeted educational interventions focused on common clinical dilemmas in STEMI patients, such as anti-coagulation, may also be of benefit. Prompt referral to allied health practitioners may allow elderly patients to be discharged home with available supports. Each of these interventions could be evaluated in separate quality improvement initiatives.

Our work must be interpreted in the context of a number of limitations. First, ours was a single tertiary care center in Ontario, Canada. As such, the specific barriers we identified may not be generalizable to other practice locations. Second, we focused on a relatively narrow clinical area, that of discharge planning post reperfusion for acute STEMI patients. We feel however that the general principals identified by our work are broadly applicable.

## Conclusions

This study was a quality improvement initiative designed to evaluate the prevalence and root causes of increased length of stay in STEMI patients. We identified patient factors, provider factors and factors related to transitions to outpatient care that influenced decisions regarding discharge. In particular, our data suggest that concerns about medication compliance, the adequacy of patient education and advanced patient age may make practitioners less likely to pursue early discharge. Future protocol based interventions to reduce length of stay post STEMI should focus on in-patient and out-patient strategies to address these unique clinical issues.

## Additional file

Additional file 1: Appendix. Zwolle score & interview questions. (DOCX 15 kb)
