# Supplementary material for: Root causes for delayed hospital discharge in patients with ST-segment Myocardial Infarction (STEMI): a qualitative analysis
Source: BMC Cardiovasc Disord. 2015 Sep 29;15:107. doi: 10.1186/s12872-015-0105-2 (PMC4589199; doi:10.1186/s12872-015-0105-2)
Supplement: Additional file 1: Appendix. — Zwolle score & interview questions. (DOCX 15 kb) [file 12872_2015_105_MOESM1_ESM.docx]

Appendix 1

| Clinical Variable | Points |
| --- | --- |
| **Killip Class**  I  II  III-IV | 0  4  9 |
| **TIMI flow**  3  2  0-1 | 0  1  2 |
| **Age**  <60  =>60 | 0  2 |
| **3 Vessel Disease**  No  Yes | 0  1 |
| **Anterior Infarction**  No  Yes | 0  1 |
| **Ischemia Time**  <4 hours  >4 hours | 0  1 |

The Zwolle score is an externally validated tool to estimate mortality at 0-2 days, 2-10 days and total 30 day mortality. Points are summed to a maximum score of 16. At a total score of less than or equal to 3, estimated mortality at 0-2 days is 0.1%, 2-10 days is 0.2%, and at 30 days is 0.5%. [5]

Appendix 2:

Cases Used in Semi-Structured Interviews

Case 1

62 year old male. Type 2 Diabetic, with hypertension who works as a construction worker.

Presents on Friday with new onset retro-sternal chest pain at work. Presented to Sunnybrook emergency department and diagnosed with inferior ST-segment elevation myocardial infarction (STEMI). Taken to cardiac cathetearization laboratory, and a 100% proximal occlusion noted in a dominant right coronary artery. No other significant flow limiting lesions. Angioplasty performed with insertion of a bare metal stent, with normal flow post procedure. Total ischemic time 6 hours. Transferred to the cardiac general ward on Saturday. No arrhythmia, no heart failure, no heart block. Echocardiogram showed moderate left ventricular dysfunction, with inferior hypokinesis. You round on the patient on Monday, and he asks you “Doctor, can I go home”

Medications: Ramipril 2. 5 mg/day, Metoprolol 25 mg PO bid, Atorvastatin 80 mg PO OD, ASA 81 mg PO OD, Ticagrelor 90 mg PO bid

On examination, blood pressure: 125/78, heart rate- 70, JVP normal, no murmurs, no cath complications

Questions:

- Would you send this patient home?
- If not, why not?
- If he asked you to go home on Sunday, what would you say?

Case 2

88 year old female, with hypertension.

Presents Monday with anterior ST-segment elevation myocardial infarction. Total ischemia time 3 hours. Angioplasty to the mid left anterior descending artery with a bare-metal stent. Good flow post stent implantation. Chronic total occlusion of the dominant right coronary artery. Moderate left ventricular dysfunction (akinetic distal anterior wall and apex). No clinical heart failure throughout the admission. Ambulating on ward with physiotherapy on Wednesday. They have no functional concerns. Her husband asks you “Can he go home today?”. Creatinine is 90 mmol/l

Medications: Bisoprolol 2.5 mg/day, Ramipril 2.5 mg/day, Atorvastatin 40 mg/day, ASA 81 mg/day, Clopidogrel 75 mg/day

On examination, blood pressure-105/62, heart rate- 70. No murmurs, no clinical signs of heart failure.

Questions:

- Would you send this patient home?
- If not, why not?

Case 3

59 year male. Dyslipidemia.

Presents Tuesday with anterior ST-segment elevation myocardial infarction. Crackles on examination in the emergency department. Proximal left anterior descending artery stented with 1 drug eluting stent with normal flow. Echocardiogram shows moderate left ventricular dysfunction with akinetic anterior wall and apex. Given 1 dose of Lasix. On Thursday he is ambulating on the cardiology general ward. He asks you, can I go home?

Medications- Ramipril 1.25 mg/day, Metoprolol 25 mg PO bid, Atorvastatin 80 mg/day, ASA 81 mg/day, clopidogrel 75 mg/day

On examination, blood pressure- 98/70, heart rate- 85, No murmurs, no clinical heart failure

Questions:

- Would you send this patient home?
- If not, why not?
